# Neuroma Treatment With the Acellular Nerve Allograft Reconstruction Technique

**DOI:** 10.7759/cureus.39567

**Published:** 2023-05-27

**Authors:** Jennifer A Bell, Collean Trotter, Daniel Gittings, Mathew Schur, Kurt M Mohty, Rachel Lefebvre, Milan Stevanovic

**Affiliations:** 1 Department of Orthopaedic Surgery, University of Southern California, Los Angeles, USA; 2 Department of Orthopaedic Surgery, University of Southern California Keck School of Medicine, Los Angeles, USA

**Keywords:** nerve relocation, human nerve allograft, nerve injury, acellular nerve allograft reconstruction, neuroma

## Abstract

Treatment of a painful neuroma is a challenging problem for both the patient and the providers. Current surgical treatment options typically include excision of the neuroma and stump relation. However, with both treatment options, patients have high rates of persistent pain and rates of neuroma recurrence. We describe two patients with neuromas treated with our acellular nerve allograft reconstruction technique. This technique involves the excision of the neuroma and bridging the proximal nerve end to the surrounding tissue with an acellular nerve allograft. Both patients had immediate resolution of their neuropathic pain that was maintained at their final follow-up. Acellular nerve allograft reconstruction is a promising treatment option for the treatment of painful neuromas.

## Introduction

Neuromas represent a painful result of nerve injury with a relatively unclear pathophysiology. Neuromas are “the inevitable, unavoidable, and biologic response of the proximal stump after it has been divided in situations where regenerating axons are impeded from re-entering the distal stump” [1]. Histologically, neuromas are characterized by nerve fibers and fascicles at multiple stages of regeneration. Fibrotic tissue, random arrangement of nerve fibers, and perineural scarring can also be present [2,3]. While the pathophysiology of neuromas remains unclear, many etiologies exist including chronic irritation, avulsion, pressure, post-amputation neuromas, inadequate repair, and trauma of the affected nerve [2,3].

Neuroma treatments have evolved over the years with the primary goal of pain relief. Generally, surgical treatment is the mainstay, but the most effective surgical method is not clear. Despite surgical treatment efforts, outcomes of neuroma treatment remain relatively inconsistent. Between 20-30% of neuromas show resistance to treatment, including surgical interventions independent of type [4-6]. Additionally, patients that receive surgical intervention often undergo repeat surgical interventions and various studies demonstrate reoperation rates of up to 65% [4,5,7,8]. More recent innovations in painful neuroma treatment methods have transitioned to the use of acellular nerve allografts (ANA) to cap proximal nerve stumps following resection. Acellular nerve grafts show promise in the treatment of symptomatic neuromas due to the inability of axons to grow past ANAs longer than 3 cm while also providing structure for organized axon growth to termination [9-11].

The purpose of this paper is to describe our acellular nerve allograft reconstruction technique for the treatment of neuromas. Our technique has been mentioned briefly in the literature; however, the technique has not been previously described prior to our previous study evaluating the treatment of saphenous neuroma following knee arthroscopy [12,13]. This technique, using a processed human nerve allograft, is thought to 1) provide a lengthy and organized environment for the regeneration of axons and 2) relocate the proximal stump to a more suitable location away from noxious stimuli.

Anatomy

The dendrites connected to the cell body continuing into an axon represent functional components of the nerve. A column of cytoplasm containing cytoskeletal elements and the matrix surrounded by a cell membrane composes the axon itself. Schwann cells encase the axons, forming a nerve fiber in both myelinated and non-myelinated conformations. From deep to superficial, the connective tissue elements of nerves include the endoneurium ensheathing the nerve fibers themselves, the perineurium, which joins fibers into fascicles, the epineurium, which encases and fills between fascicles, and the paraneurium, which allows for nerve glide. The connective tissue portions of nerves contain collagen fibrils, vascular components, and cells arranged longitudinally. The epineurium is the most abundant connective tissue element, accounting for 60-85% of the cross-sectional area of most nerves while the paraneurium contains the most vasculature [1].

Acute or chronic traumatic nerve injury may cause neuroma formation [2]. In an acute injury, such as during nerve transection, the injured stump may fail to reach the distal ends, causing disorganized axon growth and contributing to neuroma formation. Chronic injury of nerves caused by continued irritation contributes to neuroma formation through perineural scarring [2]. The perineurium is thought to act as a barrier to axon regeneration [2,14]. Damage to the paraneurium can lead to fascicular escape and cause leakage of randomly arranged axons, fibroblasts, and blood vessels into the neighboring epineurium. The proposed pathophysiology for neuromas following stretch injuries is similar. While the exact pathophysiology of neuromas is yet to be elucidated, it seems that these damaged nerves lose organization [2].

Indications/contraindications

Evaluation of a patient with a painful neuroma begins with a thorough history and physical exam. Trauma, particularly a penetrating injury, is the main cause of neuroma formation. However, neuromas can also be secondary to chronic irritation, pressure, stretch injury, crush injury, blunt trauma, or poor repair of a previous nerve injury [2,13]. Patients will either present with hyperesthesia or motor nerve changes such as weakness and muscle wasting. Four types of pain associated with neuromas include spontaneous pain, pain on pressure over the neuroma, pain on movement of adjacent joints, and painful hyperesthesia on light touch in the vicinity of the neuroma [15]. On physical exam, the patient will typically have a discrete area that is hypersensitive to light touch, a positive Tinel’s sign, and altered sensation distally [2]. Patients should undergo a trial of nonoperative interventions, including optimization of pain regimen and occupational or physical therapy [16]. For those who fail nonoperative treatment, a local anesthetic nerve block is a powerful tool to confirm the diagnosis of a neuroma for both the patient and the surgeon. In the clinic, the senior author will inject the point of maximal sensitivity on Tinel’s test with 1 to 5 cc of 1% lidocaine without epinephrine. If the patient has significant or complete relief of his or her symptoms and neuroma is the leading diagnosis, the patient is indicated for surgery. Patients should be counseled on the risk of persistent pain or recurrence of a neuroma. Indications for nerve translocation include when there is no distal stump, when the repair site is superficial and would be at risk for mechanical sensitivity, and where a repair is not possible [1].

## Technical report

Technical overview

**Figure 1:**
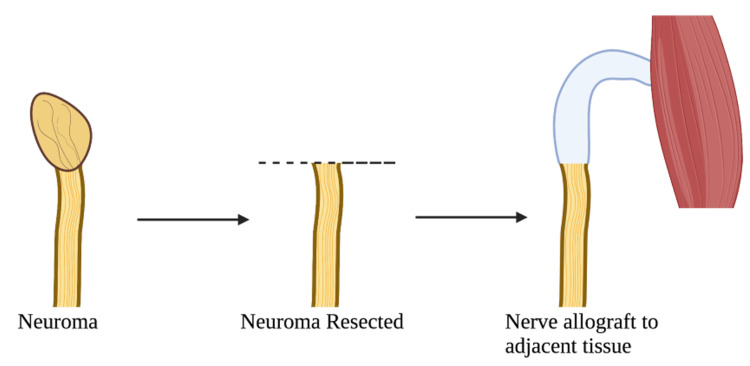
Illustration demonstrating resection of a neuroma and the acellular nerve allograft reconstruction technique Image created with Biorender.com

In the preoperative area, the point of maximal sensitivity with Tinel’s test is identified and marked to indicate the site of the neuroma. The patient is positioned appropriately to facilitate the exposure of the suspected neuroma location. The senior author prefers to use a tourniquet if possible. The appropriate superficial and deep approach to the neuroma should be utilized to dissect down to the neuroma. Once the neuroma is identified, an attempt to find the distal stump and possible nerve repair should be performed. The neuroma is then sharply resected with an 11-blade so there is only healthy nerve remaining proximally. A processed human nerve allograft is then selected to match the width of the proximal nerve stump. The typical length of the acellular nerve allograft is 5 to 7 cm. The distal end of the resected nerve and the proximal end of the nerve allograft are then loosely apposed. The distal end of the nerve allograft is placed into the surrounding soft tissue, most commonly into a muscle or subcutaneous tissue, without being under tension (Figure 1). The native nerve and nerve allograft ends are then sutured together using microsurgical instruments and a 9-0 nylon on a 100-micron needle in an interrupted fashion. Fibrin glue is then applied to the sutured ends to reinforce the coaptation. If a tourniquet was used, it is let down, and then adequate hemostasis is obtained. Appropriate closure is performed. Typically, the surgical area is immobilized for two to three weeks.

Example Case 1

**Figure 2:**
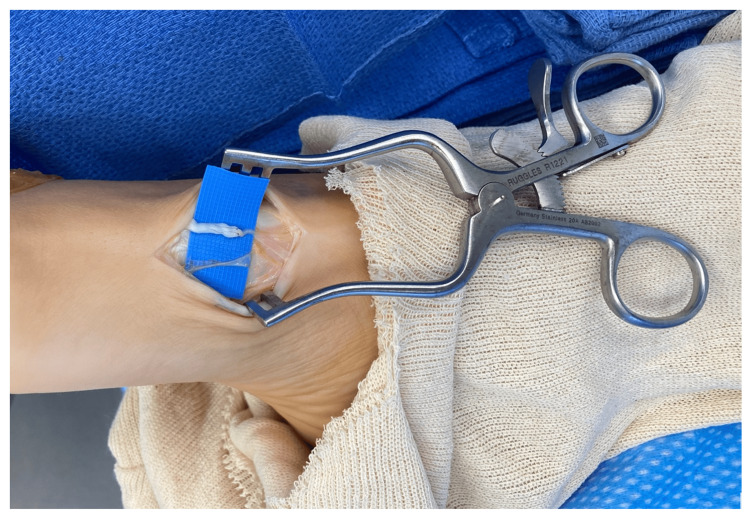
Image of right foot (toes-left, leg-right) of a patient who underwent neuroma resection and acellular nerve allograft reconstruction technique of the dorsal lateral branch of the deep peroneal nerve

The patient is a 20-year-old female who sustained a dog bite to her right dorsal foot 11 months prior. She reported persistent pain over her dorsal and medial foot. On physical exam, she had a positive Tinel’s sign over the dorsomedial foot with minimally decreased sensation distally. In the clinic, the point of maximal tenderness was injected with 3 cc of 1% lidocaine, and she experienced near-complete relief of her symptoms. Preoperatively, the point of maximal tenderness was marked. A tourniquet was placed on the right lower extremity. An incision was centered over the point of maximal tenderness. The dorsal medial and dorsal lateral branches of the deep peroneal nerve were dissected and a neuroma of the dorsal lateral branch was identified (Figure 2). The neuroma was sharply resected proximal to the neuroma. An AxoGen Avance nerve allograft, 1-2 mm x 30 mm, was then sutured to the remaining distal aspect of the dorsal lateral branch and reinforced with fibrin glue. The distal end of the allograft was then buried into subcutaneous fat and tissue. The incision was closed with a running 4-0 Monocryl suture. The patient was immobilized and remained non-weight bearing for a total of three weeks. At the first follow-up appointment at one week, she had a complete resolution of her neuropathic pain.

Example Case 2

**Figure 3:**
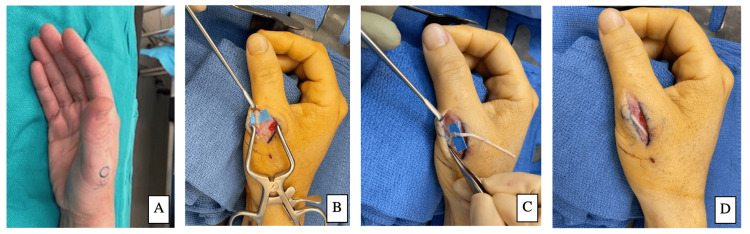
A) Pre-operative point of maximal tenderness, B) Neuroma, C) Newly resected proximal nerve co-apted to acellular human allograft, D) Nerve allograft placed tunneled to dorsal hand

The patient was a 48-year-old right-hand dominant male who sustained an approximately 3 cm transverse laceration to the dorsal aspect of his right thumb following a crush injury. The laceration was closed primarily and healed uneventfully. In the following months, he developed extreme hypersensitivity in the area of the scar. On physical exam, the patient had a positive Tinel’s sign over the scar, decreased sensation, and allodynia just distal to the laceration on the dorsal thumb. In the clinic, the patient received an injection of 1% lidocaine at the point of maximal tenderness and had a near-complete resolution of his symptoms. The patient reported his pain was disabling to his activities of daily living and desired surgical correction. On the day of surgery, the point of maximal tenderness was marked preoperatively seen in Figure 3A. A longitudinal incision was made over the dorsal thumb over the area of maximal tenderness and the neuroma of the dorsal sensory nerve was easily identified (Figure 3B). The nerve was then dissected proximally to identify healthy fascicles at which point the neuroma was excised. An acellular human AxoGen allograft measuring 1-2 mm x 50 mm (AxoGen Inc., Alachua, Florida) was then co-apted to the newly resected proximal nerve with a 9-0 nylon suture in an interrupted fashion (Figure 3C). A subcutaneous tunnel was dissected over the dorsal hand proximally and the distal end of the allograft was placed into the pocket (Figure 3D). The incision was closed with interrupted 4-0 nylon sutures, dressed in Xeroform, and the patient was placed in a thumb spica splint. At two weeks postoperatively, the patient's sutures were removed and the splint was discontinued. The patient reported complete resolution of his symptoms at the two-week follow-up and remained asymptomatic at his last follow-up visit (seven months postoperatively).

## Discussion

Various treatment options have been proposed for the treatment of end neuromas. The most common complication of this procedure, and other painful neuroma treatments, includes the persistence of the painful neuroma. Many sources report 20-30% of neuromas will continue to be painful regardless of surgical intervention [4]. Reoperation rates for painful neuromas have been reported as high as 65% [4,5,7,8]. In a meta-analysis by Poppler et al., 77% of patients had a clinically meaningful improvement regardless of surgical technique: excision and transposition, excision only, neurolysis and coverage, excision and repair, or excision and capping. Neuroma excision, followed by burying the distal nerve stump into bone, muscle, or vein, reduced pain more effectively in painful neuromas when compared to excision alone. In studies where greater than 60% of the patients had previous surgery for neuroma treatment, both excision and transposition and neurolysis and coverage had significant improvements in pain compared to excision only [4]. Rose et al. described the use of external neurolysis, followed by the insertion/coverage of the nerve stump with a nearby flap of lumbrical, abductor pollicis brevis, and adductor pollicis muscle in the treatment of neuroma in continuity [17,18].

In a previous study, we retrospectively evaluated nine patients with post-knee arthroscopy saphenous neuroma in continuity who underwent resection and reconstruction with our acellular nerve allograft reconstruction technique. At the final follow-up (average 9 months), six (66%) patients reported subjective improvements. Two of the three patients who reported no improvement had a concurrent diagnosis of chronic regional pain syndrome. However, this study was limited by the small number of patients and relatively short follow-up period.

There are many different surgical treatment options for a painful end neuroma [1,16]. Challoner et al. describe an algorithmic approach for the treatment of both end neuroma and neuromas in continuity. Common treatments for a painful end neuroma include nerve relocation, nerve capping, regenerative peripheral nerve interface (RPNI), and targeted muscle reinnervation [16]. Nerve relocation includes relocating the proximal nerve stump into deeper tissue, including muscle, bone, fat, or veins in hopes of providing an environment without mechanical irritation or risk of trauma [16]. Relocation and burying methods are limited due to the incredible resilience of axons, which often demonstrate growth beyond enclosures, contributing to subsequent pain [9,19,20]. Capping of the nerve involves covering the newly resected proximal stump with either synthetic material or biological tissue [21]. Regenerative peripheral nerve interface (RPNI) represents an additional treatment for reducing symptomatic neuroma pain in 85% of patients, and 71% had a negative Tinel’s sign post-op [22]. During an RPNI procedure, a muscle graft is harvested (brachioradialis and/or vastus lateralis in this study), sutured to, and wrapped around the proximal nerve stump following complete excision of a painful neuroma. Subsequently, the muscle-encased nerve ending is implanted into soft tissue and fixed with sutures. RPNI has additional prophylactic and therapeutic indications in cases of limb amputations [23]. Targeted muscle re-innervation (TMR) is an additional option for neuroma prophylaxis following limb loss [18,24,25]. In TMR, the proximal edge of a transected nerve (ex. median nerve) is rerouted and co-apted with a nearby nerve (ex. small branches of the musculocutaneous nerve) to innervate a smaller portion of the target muscle [23].

Acellular nerve allografts were previously evaluated as a potential nerve graft repair, however, axons were unable to cross long ANAs. With this knowledge, Hong et al. described using long ANAs as a way to provide a scaffold for axon growth and provide a controlled environment for the termination of axon growth. They evaluated this method, the ANA “cap,” in a rat model and found ANAs >0.5 cm had no regeneration in the distal end of the cap on histology [9]. The acellular nerve allograft reconstruction technique uses this concept, combined with relocation to a more suitable environment, away from possible irritants. The benefit of our technique is ANA avoids donor site morbidity and complications. Downsides include the costliness of acellular nerve allografts and not all hospitals and surgery centers have them easily accessible.

## Conclusions

We believe our acellular nerve allograft reconstruction technique is a valuable treatment option for the surgical management of painful neuromas. The benefits of our technique are two-fold, including providing an organized environment for the regenerated axon to grow as well as relocating the nerve away from the source of the irritant. While we have previously published this technique in a small cohort of patients with post-knee arthroscopy saphenous neuroma, it was a relatively small cohort with two patients who were currently being treated for chronic regional pain syndrome. However, this paper describes the use of our acellular nerve allograft reconstruction technique in various anatomic locations in patients with painful neuroma with complete relief of their symptoms. Further studies with larger cohorts and various anatomic locations will need to be performed to further evaluate long-term outcomes and identify patients at risk of persistent symptoms.
